# Coordinated Regulation of Nuclear Receptor CAR by CCRP/DNAJC7, HSP70 and the Ubiquitin-Proteasome System

**DOI:** 10.1371/journal.pone.0096092

**Published:** 2014-05-02

**Authors:** Yoav E. Timsit, Masahiko Negishi

**Affiliations:** The Pharmacogenetics Section, Laboratory of Reproductive and Developmental Toxicology, National Institute of Environmental Health Sciences, National Institutes of Health, Research Triangle Park, North Carolina, United States of America; Lund University, Sweden

## Abstract

The constitutive active/androstane receptor (CAR) plays an important role as a coordinate transcription factor in the regulation of various hepatic metabolic pathways for chemicals such as drugs, glucose, fatty acids, bilirubin, and bile acids. Currently, it is known that in its inactive state, CAR is retained in the cytoplasm in a protein complex with HSP90 and the tetratricopeptide repeat protein cytosoplasmic CAR retention protein (CCRP). Upon activation by phenobarbital (PB) or the PB-like inducer 1,4-bis[2-(3,5-dichloropyridyloxy)]-benzene (TCPOBOP), CAR translocates into the nucleus. We have identified two new components to the cytoplasmic regulation of CAR: ubiquitin-dependent degradation of CCRP and protein-protein interaction with HSP70. Treatment with the proteasome inhibitor MG132 (5 µM) causes CAR to accumulate in the cytoplasm of transfected HepG2 cells. In the presence of MG132, TCPOBOP increases CCRP ubiquitination in HepG2 cells co-expressing CAR, while CAR ubiquitination was not detected. MG132 treatment of HepG2 also attenuated of TCPOBOP-induced CAR transcriptional activation on reporter constructs which contain CAR-binding DNA elements derived from the human *CYP2B6* gene. The elevation of cytoplasmic CAR protein with MG132 correlated with an increase of HSP70, and to a lesser extent HSP60. Both CCRP and CAR were found to interact with endogenous HSP70 in HepG2 cells by immunoprecipitation analysis. Induction of HSP70 levels by heat shock also increased cytoplasmic CAR levels, similar to the effect of MG132. Lastly, heat shock attenuated TCPOBOP-induced CAR transcriptional activation, also similar to the effect of MG132. Collectively, these data suggest that ubiquitin-proteasomal regulation of CCRP and HSP70 are important contributors to the regulation of cytoplasmic CAR levels, and hence the ability of CAR to respond to PB or PB-like inducers.

## Introduction

The constitutive active/androstane receptor (CAR) is a member of the xenobiotic-sensing nuclear receptor, functioning as a ligand-activated transcription factor capable of regulating the expression of genes involved in the metabolism of both xenobiotics and endogenous chemicals produced in the organism [1], [2]. It was identified in the late 1990s as the principal mediator of the induction by barbiturates such as phenobarbital (PB) of the human cytochrome P450 *2B6* (*CYP2B6*), the rat *CYP2B1*, and the mouse *Cyp2b10* genes [3]–[7]. Subsequently, CAR has been found to play an important role in metabolic homeostasis and disease. For instance, we have established a role for CAR in PB-induced hepatocellular carcinoma using *CAR*-null mice [8], [9], and also in the regulation of thyroid hormone synthesis during liver regeneration [10]. Currently, ongoing efforts to understand CAR's role in physiology and disease continue to identify new pathways and mechanisms by which CAR exerts its effects upon stimulation by exogenous and endogenous activators.

While CAR's role in disease and physiology is being established, there are aspects to CAR signaling which remain unclear. Firstly, while CAR is shown *in vivo* to be localized to the cytoplasm in liver [3], [11], it is not understood why this localization becomes deregulated in cell lines [11], which renders them unsuitable to accurately model CAR's signaling and function. Second, no physiological ligand(s) for CARhave been identified, although specific chemicals have been found to bind to CAR such as the inverse agonist androstanol [12], the potent activator of mouse CAR (mCAR) TCPOBOP [13], and the activator of human CAR (hCAR) 6-(4-chlorophenyl)imidazo[2,1-b][1], [3]thiazole-5-carbaldehyde *O*-(3,4-dichlorobenzyl)oxime (CITCO) [14]. TCPOBOP in particular has unique structural characteristics that make it a potent mCAR activator [15]. Third, CAR displays species differences in the kinds of chemicals able to activate CAR [1] in spite of a 74–79% identity between human and rodent CAR [16], [17], which presents a challenge to precisely define how CAR may be activated. And lastly, new questions regarding CAR signaling have emerged from observations of its localization to the cell membrane. CAR localizes to the cell membrane of mouse liver hepatocytes [18] and is capable of cross-talking with growth factor signaling (e.g., EGF) at the level of Mek1/2 [19] and through mediation by RACK1 [20], a scaffold present in receptor- and intracellular-associated protein complexes [21]. As well, CAR interacts with the protein phosphatase PPP1R16A (R16A) at the membrane, implicating a mechanism for CAR activation in the absence of CAR activators [22]. The biological significance of these identified pathway interactions is only beginning to be understood, opening new insights into CAR signaling within the cell.

With a growth of data characterizing pathway interactions at the membrane, efforts continue to improve understanding of mechanisms by which CAR's subcellular distribution is regulated. An important step forward was the identification of protein phosphatase PP2A forming part of the CAR cytoplasmic regulatory complex that functions in CAR release and translocation [23]. Next was the discovery of the “CAR cytoplasmic retention protein” (abbreviated as CCRP) as a CAR cytoplasmic chaperone that also mediates CAR interaction with HSP90 [23], [24]. Such interactions influence the ability of CAR to translocate among the various subcellular compartments, and consequently impacting CAR-mediated transcriptional activity. However, the events that result in release of CAR from the cytoplasmic protein complex to engage with partner proteins at the cell membrane and those in the cytoplasm, such as Rho-guanine nucleotide exchange factor epithelial cell-transforming gene 2 (ECT2) [25] and GADD45β [26], [27], remain unclear. Aside from CAR dephosphorylation by PP2A, shown to be a critical requirement for translocation of CAR from the cytoplasm to the nucleus [28], not much else is known regarding CAR's dissociation from its cytoplasmic complex.

In light of a large body of evidence for ubiquitination regulating steroid and nuclear receptor function [1], [29], [30], as well as in RTK signaling [31], [32], we embarked to determine whether ubiquitination plays a role in CAR signaling. As noted above, CCRP functions to stabilize CAR in the cytoplasm when overexpressed. Hence we hypothesized CCRP as a target for ubiquitination, thus serving as a regulatory checkpoint for CAR by the ubiquitin-proteasomal system. The results presented here provide first evidence of the contribution of the ubiquitin-proteasomal system in regulating CAR levels in the cytoplasm. Moreover, a novel interactor of CAR, HSP70, has been identified as a component of the CAR cytoplasmic complex. The results presented here provide novel insights into the molecular mechanisms regulating CAR cytoplasmic retention and its release from its complex upon treatment with PB and PB-like chemical inducers.

## Materials and Methods

### Reagents

The proteasome inhibitor MG132 was purchased from Calbiochem (San Diego, CA), and TCPOBOP was purchased from Sigma (St. Louis, MO). Dual Luciferase kits were purchased from Promega Corp. (Madison, WI). Minimal essential media and L-glutamine were purchased from Invitrogen Corp. (Carlsbad, CA), and penicillin/streptomycin was purchased from Sigma. Fugene transfection reagent was purchased from Roche Diagnostics GmbH (Indianapolis, IN), and Lipofectamine 2000 was purchased from Invitrogen.

### Plasmids

The expression plasmid pCW7-Myc-Ub was generously provided by Dr. Andrew Wallace, Department of Environmental and Molecular Toxicology, North Carolina State University. Mouse CAR in pcDNA3.1/V5-His, mouse CCRP, −1.8 kb-luciferase reporter plasmid, (NR1)_6_-tk-luciferase reporter plasmid, and C-terminal FLAG-tagged CAR in pCR3 have been described elsewhere [6], [24], [28], [33].

### Cell Culture

Cell were maintained in minimal essential medium (MEM) supplemented with 10% fetal bovine serum, L-glutamine (1 mM final concentration), penicillin (100 U/ml final concentration), and streptomycin (100 µg/ml final concentration). Cells were plated in 10-cm plastic plates at 5×10^6^ cells per plate. Transfections were carried out 24 hr after plating using Fugene (Roche) according to the manufacturer's instructions. 24 hours after transfection, cells were treated with DMSO, TCPOBOP alone, MG132 alone, or TCPOBOP and MG132 in serum-free media at a final 0.1% concentration DMSO (final v/v). For heat shock experiments, cells were incubated at 42°C for 1 hour prior to chemical treatment. Following chemical treatment, cells were washed once in pre-warmed PBS and scraped into 5 ml pre-warmed PBS. Cells were then pelleted and resuspended in Buffer A (10 mM HEPES pH 7.6, 1.5 mM MgCl_2_, 10 mM KCl, 20 mM sodium molybdate (Na_2_MoO_4_), 1 mM dithiothreitol, and 0.3% Nonidet P-40) containing Complete Mini protease inhibitor cocktail (Roche) and 0.2 mM PMSF. For experiments to detect ubiquitinated protein, 5 mM N-ethylmaleimide (NEM) was also included in Buffer A. Cells were homogenized in a glass homogenizer (pestle B) and cell cytosolic and nuclear fractions prepared as described previously [24]. Briefly, homogenates were spun at 4000×*g* to pellet nuclei, and supernatants were transferred and spun at 17,800×*g*. Clarified supernatants were collected to obtain the cytosolic fraction and stored at −70°C until analysis. The pelleted nuclei were washed once in Buffer A, followed by wash in Buffer A not containing Nonidet P-40, and then suspended in nuclear extraction buffer (10 mM HEPES pH 7.6, 0.1 mM KCl, 3 mM MgCl_2_, 0.1 mM EDTA, 1 mM Na_3_VO_4_, and 1 mM dithiothreitol) supplemented with Complete Mini protease inhibitor cocktail (Roche) and 0.2 mM PMSF. NaCl was added to a final concentration of 0.4 M. Nuclear extraction was carried out for 1 h at 4°C on a Nutator shaker, followed by centrifugation at 38,0000 rpm in a Beckman Tabletop ultracentrifuge for 30 min. Supernatants were collected to obtain the nuclear extract fraction and stored at −70°C until analysis. Protein concentrations were determined using the Bradford protein reagent (Bio-Rad, Hercules, CA) [34].

### Western Blotting and immunoprecipitation analysis

Whole cytosolic and nuclear extract samples were prepared in 1X (final) LDS Nupage buffer (Invitrogen), heated for 10 min at 70°C, and then resolved by SDS-PAGE using Nupage pre-cast gradient bis-acrylamide gels (4–12%) or 10% bis-acrylamide gels (Invitrogen). After gel electrophoresis, resolved proteins were transferred onto Immobillon-P PVDF membranes (Millipore, Danvers, MA) using a Hoeffer semi-dry transfer apparatus. After protein transfer, membranes were blocked in TBST buffer (10 mM Tris pH 7.4, 0.1% Tween-20) containing 5% non-fat milk powder (Santa Cruz Biotechnology, Santa Cruz, CA). Membranes were then probed with antibody in TBST containing 5% milk powder. The following antibodies were used for immunoblotting: HRP-conjugated anti-V5 and non-HRP-conjugated anti-V5 (Invitrogen), both at 1∶5000 dilution; HRP-conjugated anti-FLAG and non-HRP conjugated anti-FLAG (Sigma) at 1∶1000 dilution; anti-Myc (clone 9E10, Covance, Emeryville, CA) at 1∶1000 dilution; and HRP-conjugated anti-Myc (Upstate Biotechnology-Millipore) at 1∶1000 dilution. For immunoprecipitation analysis, 1 µg antibody per 125 µg cytosolic protein was used and all incubations were performed in Buffer A supplemented with Complete-Mini protease inhibitor cocktail (Roche), and PMSF. To detect ubiquitinated CCRP and CAR, 5 mM N-ethylmaleimide (NEM), 1% (v/v) sodium deoxycholate, and 0.1% SDS was supplemented to Buffer A, with sodium deoxycholate and SDS added to dissociate interacting proteins. Normal mouse IgG (1 µg, Santa Cruz) was used for control immunprecipitations. Prior to immunoprecipitation, cytosols were pre-cleared with protein G-agarose resin in the same buffer above for 1 h at 4°C. Samples were nutated at 4°C, after which 40 µl of protein G-agarose (50% slurry) was added, followed by further incubation at 4°C. All precipitated immune complexes were centrifuged at 17,800×*g*, then washed twice in IP wash buffer (10 mM Tris pH 7.4, 150 mM NaCl, 1% Triton X-100, 1 mM EDTA, 1 mM EGTA, 0.5% NP-40) supplemented with Complete Mini protease inhibitor cocktail, 0.2 mM sodium orthovanadate, 0.2 mM PMSF, 1% (v/v) sodium deoxycholate, 0.1% SDS. For detection of ubiquitinated protein, NEM was supplemented in the wash buffer. Two additional wash cycles were performed using IP wash buffer without dexycholate and SDS. Pelleted immune complexes were then resuspended in 4X LDS containing 5% β-mecaptoethanol, heated at 70°C for 10 min, and then subjected to immunoblotting analysis as described above.

### Luciferase reporter assays

Cells were plated in 24-well plates at a density of approximately 250,000 cells per well. 24 h after plating, cells were transfected using Lipofectamine 2000 as per the manufacturer's instructions. The *Renilla* construct phRL-tk-luc (Promega) was used as a normalization control for transfection efficiency. Cells were then treated for 24 h and then lysed in Passive Lysis Buffer (Promega). Firefly luciferase and *Renilla* luciferase activities were assessed using the Dual-Luciferase Assay Kit (Promega) with measurements obtained using a 96-well plate format luminometer (Turner Biosystems, Sunnyvale, CA). All data are presented as mean ± SD from triplicate determinations of each treatment group.

## Results

### TCPOBOP treatment causes concomitant reduction of both CAR and CCRP

**Figure 1:**
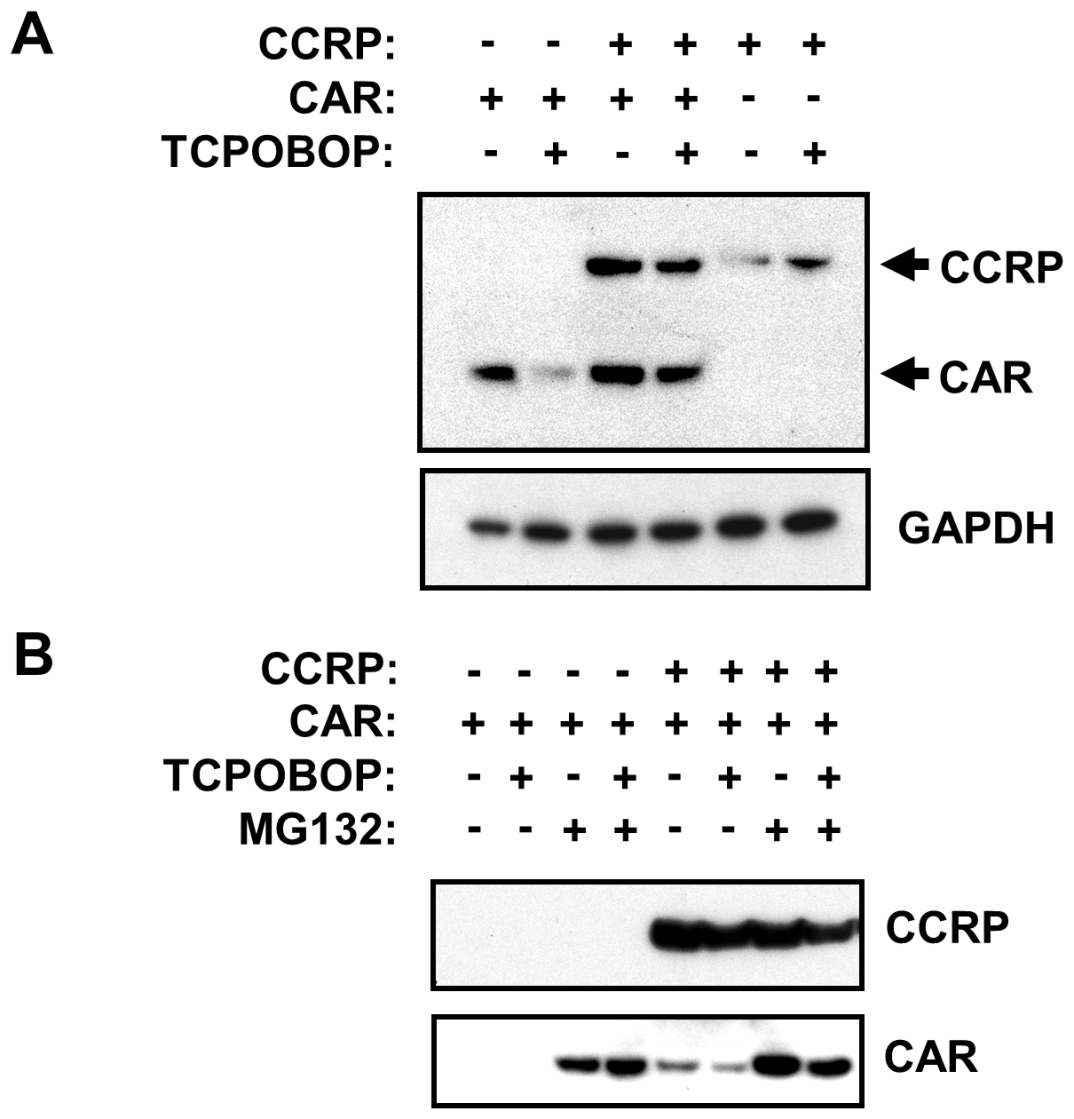
Stabilization of CAR in the cytoplasm. (A) HepG2 cells were transfected with V5-tagged mouse CAR (pcDNA3.1/V5-His-mCAR-V5, 3 µg) and mouse CCRP (pcDNA3.1/V5-His-mCCRP, 0.3 µg), and 18 hours later transfected cells were treated with DMSO (0.1% v/v) or TCPOBOP (250 nM) for 24 hr. Cells were then harvested and cytosolic extracts prepared and subjected to immunoblotting analysis with anti-V5 antibodies. (B) HepG2 cells were transfected with V5-tagged CCRP (pcDNA3.1-mCCRP-V5, 0.3 µg) or empty vector (pcDNA3.1-V5) combined with FLAG-tagged mCAR (pCR3-mCAR-FLAG, 3 µg).18 hours after transfection, cells were treated with DMSO (0.1% v/v), TCPOBOP (250 nM in 0.1% DMSO, final concentrations), MG132 (5 µM in 0.1% DMSO, final concentrations), or TCPOBOP combined with MG132 for 24 hr. Cells were then harvested and cytosolic extracts prepared and subjected to immunoblotting analysis with anti-V5 antibodies (upper panel) and with anti-FLAG antibodies (lower panel). Results shown are representative of three independent experiments.

It had been shown previously that CCRP overexpression stabilizes CAR in the cytosol of HepG2 cells, and that TCPOBOP treatment is less efficacious to cause nuclear translocation of CAR [24]. These findings were based on assessment of mCAR protein levels; however, CCRP protein levels upon TCPOBOP treatment were not ascertained therefore we proceeded to determine the effect of TCPOBOP on both CCRP and CAR. HepG2 cells were co-transfected with V5-tagged CCRP and V5-tagged CAR, and treated with DMSO or TCPOBOP (Fig. 1A). For controls, cells were co-transfected with empty vector and mCAR, or were co-transfected with empty vector and CCRP, and then treated with DMSO or TCPOBOP. As revealed by immunoblotting analysis of the cytosolic fraction of cells using an anti-V5 antibody that simultaneously detects V5-tagged CAR and CCRP, the level of mCAR in the cytosol was increased in cells co-expressing CCRP (Fig 1A, *lane 3 versus lane 1*). Also consistent with the previous study, TCPOBOP treatment gave a less efficacious decrease of cytosolic mCAR in the presesnce of overexpressed CCRP. However, in these cells, cytosolic CCRP was also decreased slightly with TCPOBOP treatment, which was absent in cells expressing V5-tagged CCRP in the absence of V5-tagged mCAR. This result suggests that TCPOBOP not only stimulates CAR nuclear translocation, but also can induce a decrease in cytosolic CCRP. As CCRP is not detectable in nuclear extracts ([24] and data not shown), this observation would be consistent with CCRP being degraded, possibly by a ubiquitin-proteasomal-dependent mechanism, upon activation of CAR.

To test whether the ubiquitin-proteasomal pathway regulates CCRP levels, HepG2 cells were co-transfected with V5-tagged CCRP and FLAG-tagged CAR [28], which in reporter experiments responds to TCPOBOP to induce transcriptional activation like the V5-tagged CAR expression construct. After transfection, cells were treated with TCPOBOP, alone or in combination with the proteasome inhibitor MG132 (5 µM). Immunoblotting analysis of the cytosolic extracts using anti-FLAG and anti-V5 antibodies (Fig. 1B and Fig. S1) reveals, firstly, that in cells overexpressing V5-tagged CCRP, the level of FLAG-tagged CAR was increased (Fig. 1B, *lanes 1 vs 5* and Fig. S1, *lanes 1 vs 4*) recapitulating the results shown in Fig. 1A. Moreover, TCPOBOP treatment reduces CAR levels, which is more apparent when CCRP is overexpressed (Fig. 1B, *lanes 6 vs 5* and Fig. S1, *lanes 5 vs 4*). In contrast, MG132 treatment substantially elevated CAR levels regardless of CCRP overexpression (Fig. 1B, *lanes 3 and 7*, and Fig. S1, *lanes 6 vs 3*), however CAR was most elevated with the combination of CCRP overexpression and MG132 treatment (Fig. 1B, *lanes 7 vs 3* and Fig. S1, *lanes 6 vs 3*). TCPOBOP was not effective at reducing cytosolic CAR levels in the presence of MG132 (Fig. 1B, *lanes 4 vs 3* and *lanes 8 vs 7*) indicating that CAR was unable to translocate to the nucleus. The apparent reduction in cytosolic CAR (Fig. 1B, *lanes 8 vs 7*) by TCPOBOP in the presence of MG132 is attributed to CCRP being expressed at a lower expression of CCRP. Taken together, these observations strongly suggest a role of the ubiquitin-proteasome system for stabilization of cytosolic levels of CAR, and this might be mediated by CCRP.

### CCRP, but not CAR, is ubiquitinated upon TCPOBOP treatment

Two possible mechanisms may explain the stabilization of CAR with proteasomal inhibition by MG132 treatment. The first is that CAR is directly ubiquitinated and degraded by the proteasome, and that CCRP blocks ubiquitination to stabilize enough CAR that can be activated by TCPOBOP. Alternatively, CCRP is ubiquitinated, and by inhibiting the proteasome CCRP is not degraded and thereby stabilizing CAR. TCPOBOP may activate CAR by stimulating CCRP ubiquitination resulting in it being degraded, thereby releasing CAR from the cytosolic complex to translocate to the nucleus. In order to determine whether CAR or CCRP is ubiquitinated, we transiently co-expressed in HepG2 cells V5-tagged CCRP, Myc-tagged ubiquitin, and FLAG-tagged CAR, and treated cells as follows: DMSO, MG132 alone, or MG132 with TCPOBOP. Cytosols were prepared after 24 hour treatment and were subjected to immunoprecipitation analysis to detect ubiquitinated CAR and CCRP. From the same cytosolic extract, anti-V5 and anti-FLAG immunoprecipations were performed, and immunoprecipitates were probed with anti-Myc antibodies to detect ubiquitin, anti-V5 antibodies to detect CCRP, and anti-FLAG antibodies to detect CAR. The use of Myc-tagged ubiquitin follows the commonly-employed approach of using tagged forms of ubiquitin to increase sensitivity for detecting ubiquitinated proteins [35], [36]. To control for the specificity of the anti-Myc antibodies used for detecting Myc-tagged ubiquitin, transfection of HepG2 cells without the inclusion of Myc-ubiquitin expression plasmid was performed in parallel. Cytosols prepared from these cells were analyzed at the same time and in the same manner as the cytosols obtained from cells where Myc-ubiquitin was expressed. It is important to note that inclusion of such controls is to ensure specificity in the detection of Myc-ubiquitin-tagged proteins. However, interpretation will be based primarily on analysis of cytosolic extracts prepared from cells transfected with all three expression plasmids and comparing the effect of MG132 versus MG132 plus TCPOBOP treatment

**Figure 2:**
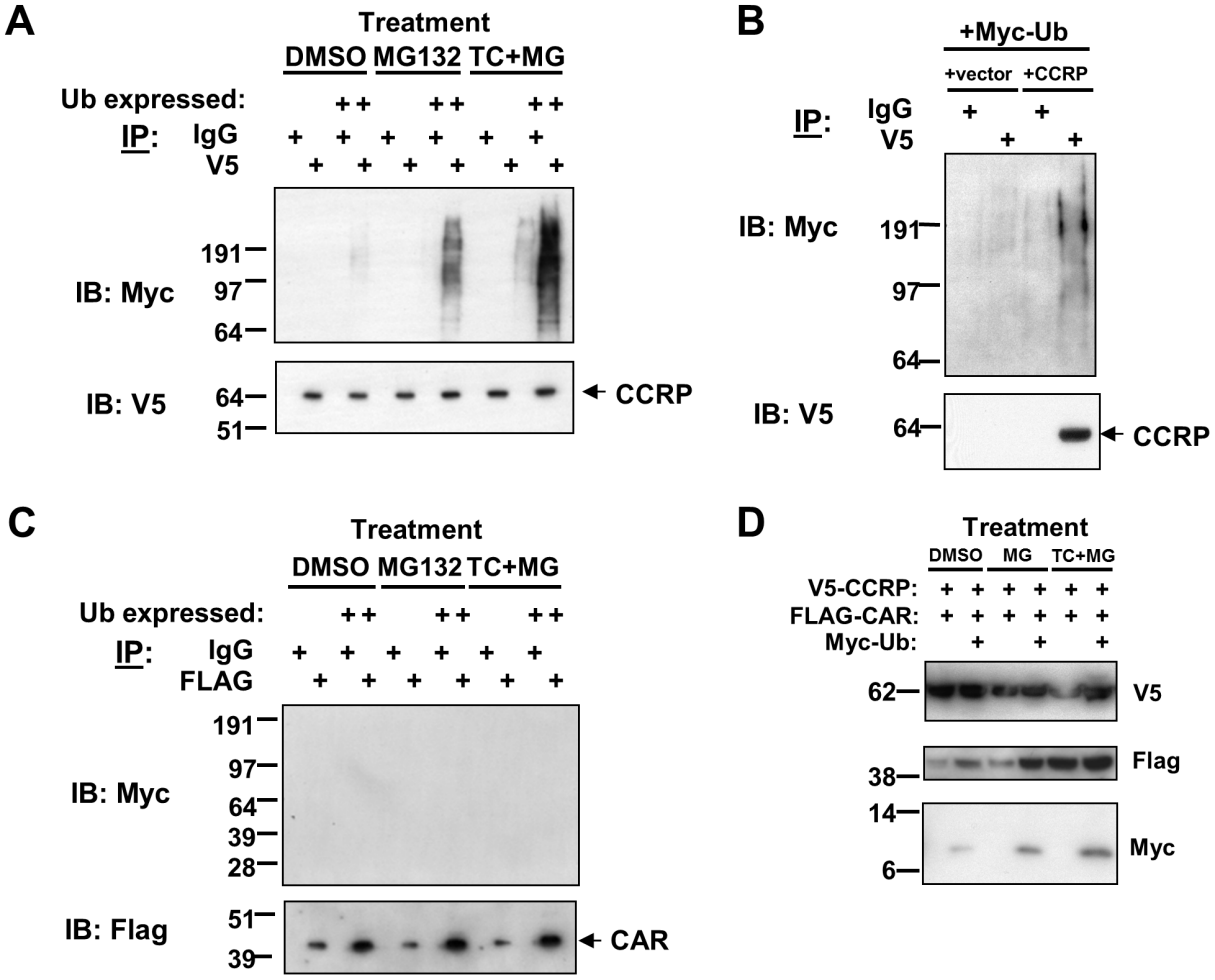
CCRP is ubiquitinated upon TCPOBOP treatment. Various combinations of the following plasmids were transfected into HepG2 cells 18(cell density 5×10^6^ cells/10 cm plate): pcDNA3.1/V5-His-mCCRP (3 µg), pCR3-mCAR-FLAG (3 µg), and pCW7-Myc-Ub (3 µg). 24 hr after transfection, cells were treatment with DMSO (0.1% v/v), MG132 (5 µM in 0.1 1% DMSO, final concentrations) or MG132 (5 µM) in combination with TCPOBOP (250 nM dissolved in 0.1% DMSO, final concentrations). At the end of treatment, cells were harvested, cytosolic extracts prepared, and immunoprecipitation analysis was performed to detect ubiquitinated CCRP and CAR. Results shown are representative of three independent experiments. (A) Cells were transfected pcDNA3.1/V5-His-mCCRP (3 µg) and pCR3-mCAR-FLAG (3 µg) with or without pCW7-Myc-Ub (3 µg). Immunoprecipitation using anti-V5 antibodies or anti-IgG (as control) was performed, followed by immunoblotting analysis using anti-V5 and anti-Myc antibodies. (B) As a control for detecting ubiquitinated CCRP, extracts derived from cells cotransfected with empty vector or pcDNA3.1/V5-His-mCCRP and pCW7-Myv-Ub were subjected to immunoprecipation then immunoblotting analysis in (A). (C) Extracts were also subjected to imunoprecipitation analysis using anti-FLAG antibodies or anti-IgG (as control), followed by immunoblotting analysis using anti-FLAG and anti-Myc antibodies. (D) Immunblotting results of extracts used in the immunoprecipitation experiments, using anti-V5, anti-FLAG, and anti-Myc antibodies.

The results reveal that CCRP, but not CAR, is ubiquitinated and that TCPOBOP increases the level of CCRP ubiquitination (Fig. 2). Western blotting of V5-purified proteins using anti-Myc antibodies detected band shifts consistent with ubiquitinated CCRP. These shifted bands appear above 64 kDa (Fig. 2A, *upper panel*), the molecular weight of V5-tagged CCRP as revealed by western blotting analysis of the same immunoprecipitates using anti-V5 antibodies (Fig. 2A, *middle panel*). This result provides first evidence of CCRP ubiquitination in HepG2 cells. Interestingly, CCRP ubiquitination was detected at a low level in cells treated with DMSO, suggesting that CCRP undergoes a basal level of ubiquitination. However, MG132 treatment alone resulted in a dramatic increase in the level of CCRP ubiquitination, and this was further increased by co-treatment of MG132 and TCPOBOP. This enhancement is CAR-dependent, as the level of CCRP ubiquitination in the absence of CAR did not increase (data not shown). Equal amounts of V5-tagged CCRP was purified in all experimental samples, hence the increased levels of CCRP ubiquitination is not due to an increase in the amount of immunoprecipitated CCRP. Some background ubiquitinated protein was detected by anti-Myc immunoblotting (Fig. 2A, *upper panel*) of IgG-control immunoprecipitates which did not contain V5-tagged CCRP (Fig. 2A, *lower panel*) as well as in cells not expressing CCRP (Fig. 2B, *upper panel*). Nevertheless, the band intensity for the shifted bands above 64 kDa was clearly elevated above background when CCRP is overexpressed (Fig. 2A and 2B, *upper panels*). To exclude the possibility of non-specific protein binding of the anti-Myc antibodies, cells not expressing Myc-tagged ubiquitin but expressing V5-tagged CCRP and FLAG-tagged CAR were included in the analysis. The lack of any Myc immunoreactivity in these V5 immunoprecipitates (Fig. 2A) confirms the specificity of the anti-Myc antibodies. Collectively, these results indicate that CCRP is ubiquitinated, and that CCRP ubiquitination is increased upon TCPOBOP treatment.

To determine if CAR is ubiquitinated, anti-Myc western blotting of FLAG-purified proteins obtained from the same cytosolic extracts used in V5 immunoprecipitations was performed. No shifted bands were detected above 42 kDa (Fig. 2C, *upper panel*), the molecular weight of FLAG-tagged CAR as detected by anti-FLAG immunoblotting of the precipitates (Fig. 2C, *lower panel*). This anti-FLAG immunoblot also demonstrates that equal amounts of FLAG-tagged CAR was purified in the experimental samples where Myc-tagged ubiquitin was expressed. Thus, under the current experimental conditions, we are unable to detect ubiquitinated CAR.

Lastly, to exclude the possibility that the increased amount of ubiquitinated CCRP in cells treated with both MG132 and TCPOBOP compared to cells treated with MG132 alone was not due to a similar increase in expression of Myc-tagged ubiquitin, western blotting of the cell cytosolic was performed to determine the expression levels of V5-CCRP, FLAG-CAR, and Myc-ubiquitin. In cells treated with both MG132 and TCPOBOP, the level of Myc-ubiquitin did increase slightly compared to cells treated with MG132 alone, while CAR and CCRP expression levels were equal between treatments. However, this increase was much less than the extent of increase in the amount of ubiquitinated CCRP detected in anti-V5 immunoprecipitates upon treatment with MG132 and TCPOBOP compared to treatment with MG132 alone. This further strengthens our interpretation of the data presented here that CCRP is ubiquitinated, and that TCPOBOP increases CCRP ubiquitination in a CAR-dependent manner. Moreover, in attempts at detecting CCRP ubiquitination in an *in vitro* assay, approximately 10 kDa shifts in CCRP protein had been detected (data not shown), supporting the results obtained in cells.

### Proteasomal inhibition attenuates CAR transcriptional activation in HepG2 cells

As proteasomal inhibition with MG132 increases the cytosolic level of CAR in HepG2 cells, we then hypothesized that transcriptional activation by CAR would be enhanced with the increased level of cytosolic CAR that can then translocate to the nucleus and initiate transcription. To assess CAR transcriptional activity, two reporter constructs were used in experiments in HepG2 cells. The first contains the −1.8-kb upstream fragment of the *CYP2B6* gene (-1.8-kb-luc) that includes the phenobarbital-response enhancer module (PBREM, at −1732/−1685 bp), and the second contains five repeats of the NR1 CAR-binding motif fused to tk-luc [(NR1)_5_-tk-luc]. For CAR expression, a C-terminal V5-tagged CAR expression construct was used in transfections; for negative control samples, cells were transfected with an empty vector (pcDNA3.1) in place of the CAR plasmid. Transfected cells were subject to the chemical treatments as follows: DMSO, TCPOBOP alone, MG132 alone, and both TCPOBOP and MG132. After 24 hour treatment cells were lyzed and luciferase activity was measured and normalized to *Renilla* luciferase activity produced by co-expression of *Renilla* luciferase.

**Figure 3:**
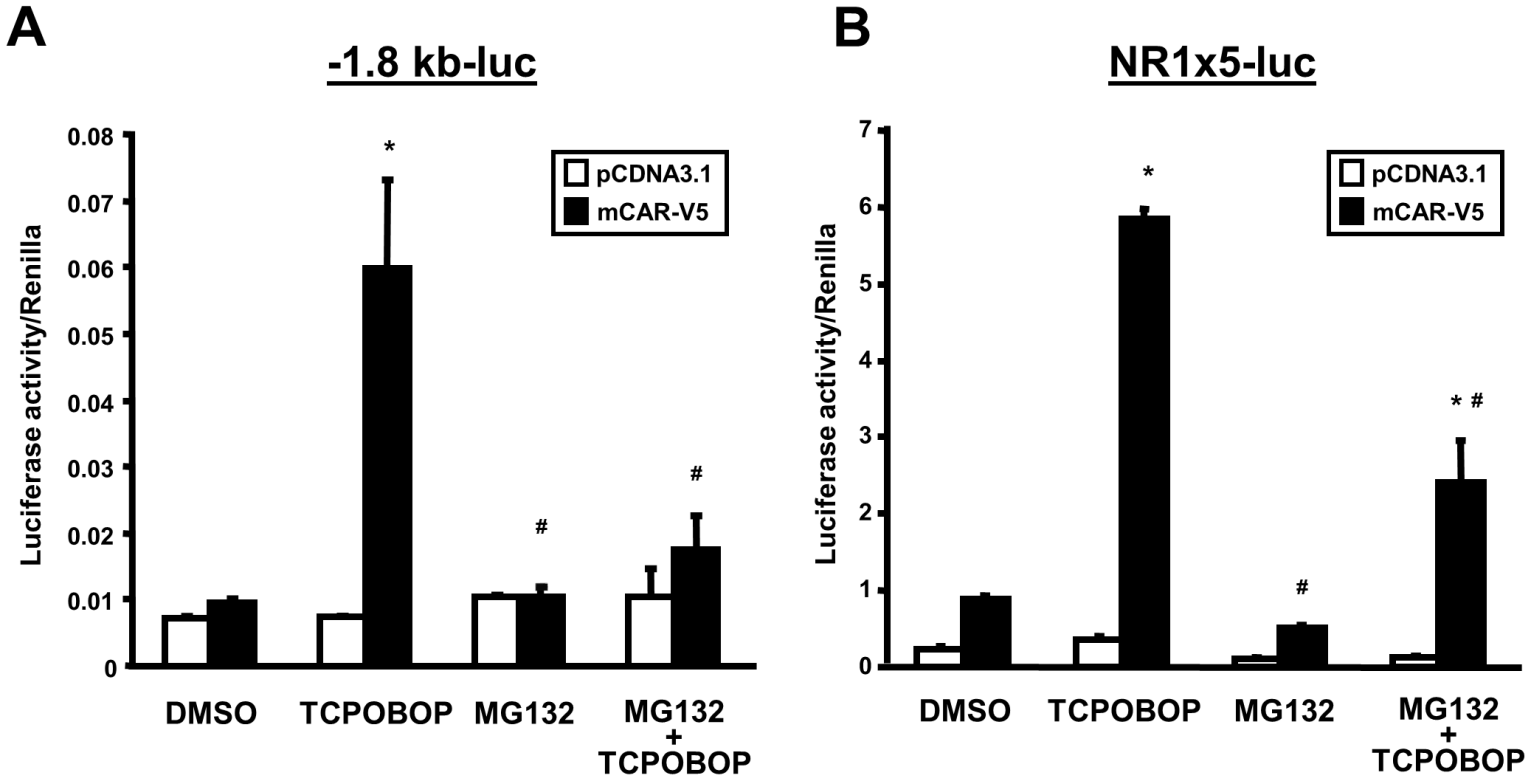
Proteasomal inhibition attenuates TCPOBOP-induced CAR transcriptional activation in HepG2 cells. Cells were cotransfected 18/V5-His-mCAR or empty vector combined with either the luciferase reporter plasmid containing −1.8 kb upstream region of the *CYP2B6* gene promoter (−1.8-kb-luc) (A) or the (NR1)_5_-tk-luciferase [(NR1)_5_-tk-luc] reporter plasmid (B). The *Renilla* construct (phRL-tk) was also co-transfected as a normalization control. Cells were then treated with DMSO (0.1% v/v), TCPOBOP (250 nM dissolved in 0.1% DMSO, final concentrations), MG132 (5 µM in 0.1 1% DMSO, final concentrations), or MG132 plus TCPOBOP, after which cells were harvested and luciferase activity measured. Luciferase activity of each sample was normalized to Renilla activity, and expressed as means ± SD of triplicate determinations. Results shown are representative of three independent experiments. *Significantly different (*p*<0.0001) compared to DMSO (+mCAR-V5); ^#^significantly different (*p*<0.0001) compared to TCPOPOP (+mCAR-V5), based on one-way ANOVA with post-hoc Tukey multiple comparisons test.

Treatment of transfected cells with TCPOBOP alone caused a six-fold or eight-fold increase in luciferase activity over DMSO-treated cells with, respectively, the −1.8-kb-luc (Fig. 3A) and (NR1)_5_-tk-luc reporter (Fig. 3B) constructs. This induction was absent when CAR was not co-expressed, indicating that when expressed CAR will transactivate with TCPOBOP treatment. MG132 treatment did not induce luciferase activity in cells expressing CAR; in cells not expressing CAR, MG132 treatment also had a minimal effect on the basal activity of the reporters. However, while TCPOBOP did increase both reporter activities in CAR-expressing cells in the presence of MG132, the activities were increased only approximately two-fold. Hence, MG132 attenuated TCPOBOP-induced transactivation for both reporter constructs Thus, in spite of the effect of MG132 of increasing CAR expression in the cytosol, this elevation did not result in enhancement of TCPOBOP-stimulated CAR transcriptional activity. These results therefore suggest that proteasomal inhibition results in retention of CAR in the cytosol. In light of our findings that TCPOBOP increases CCRP ubiquitination, proteasomal inhibition would prevent its degradation hence favoring retention of CAR in the cytoplasm, presumably in its cytoplasmic complex.

### Elevation in cytoplasmic CAR levels by MG132 is accompanied by increases in levels of heat-shock proteins

To further explore our hypothesis that proteasome inhibition stabilizes the cytosolic CAR complex, we evaluated the effect of MG132 on non-signaling components (i.e. excluding PP2A) of the CAR cytosolic complex: CAR, CCRP, and HSP90. In light of the role of HSP70 in steroid receptor function [37], [38] and based on previous reports of proteasome inhibitor-induced elevation of HSP70 in HepG2 cells [39], [40], this heat shock protein was also included in the analysis. Moreover, HSP60 was also included as the proteasome inhibitor *N*-acetyl-leucyl-leucyl-norleucinal (ALLN) does not alter HSP60 levels in HepG2 cells [40].

**Figure 4:**
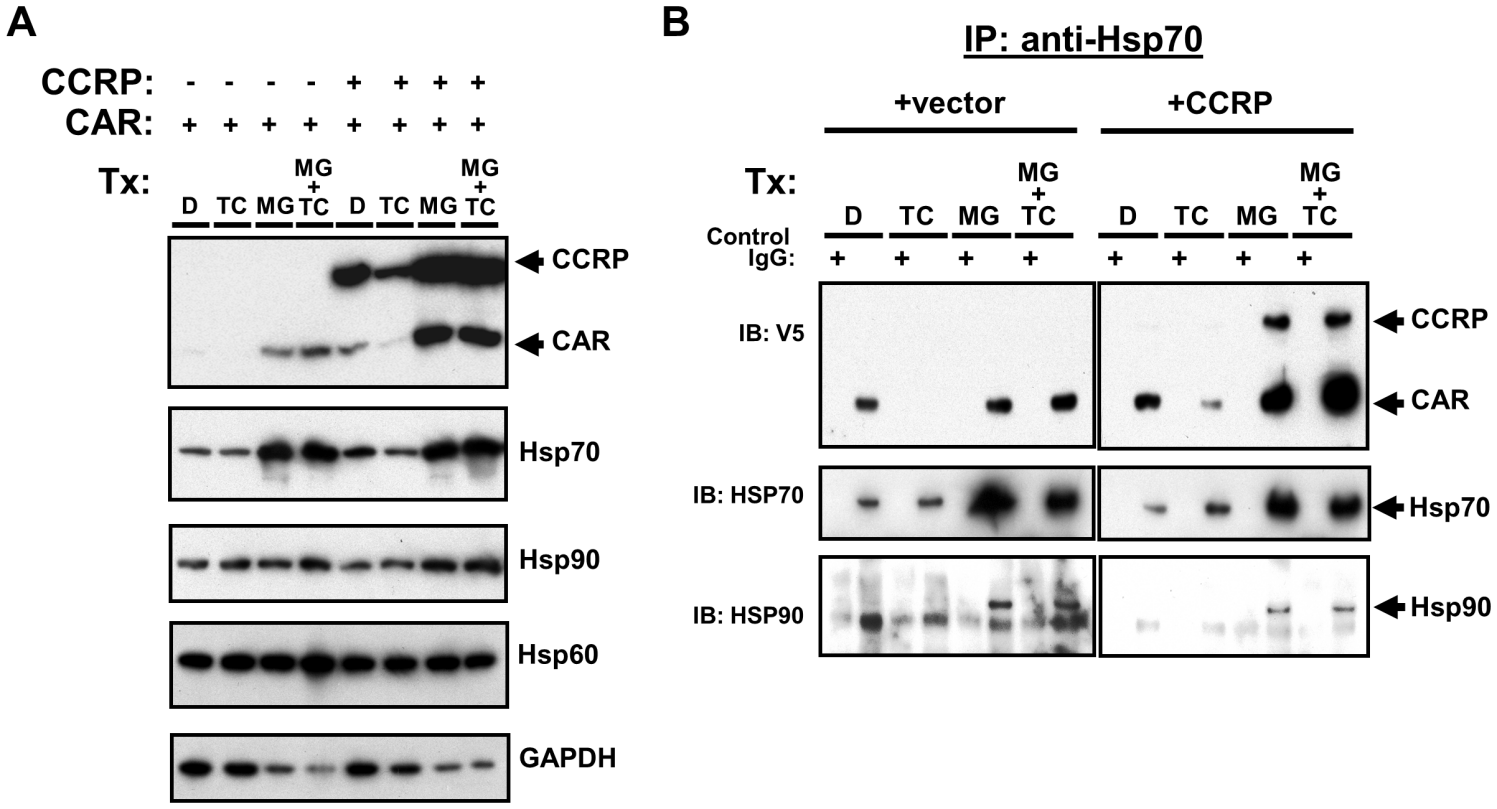
Increased levels of cytoplasmic CAR by proteasomal inhibition correlates with an increase in HSP70 levels. Results shown are representative of three independent experiments. (A) HepG2 cells were transfected with V5-tagged mouse CAR (pcDNA3.1/V5-His-mCAR, 3 µg) and mouse CCRP (pcDNA3.1/V5-His-mCCRP, 0.3 µg), and 18 hours later transfected cells were treated for 24 hr with DMSO (0.1% v/v), TCPOBOP (250 nM in 0.1% DMSO, final concentrations), MG132 (5 µM in 0.1% DMSO, final concentrations), or TCPOBOP combined with MG132. Cells were then harvested and cytosolic extracts prepared and subjected to immunoblotting analysis with antibodies against the indicated proteins. (B) Extracts analyzed in (A) were subjected to immunoprecipitation analysis using anti-V5 or anti-IgG (as control) antibodies was performed, followed by immunoblotting analysis using anti-V5 (top panel), anti-HSP70 (middle panel), and anti-HSP90 (bottom panel) antibodies.

To evaluate the effects of MG132 on CAR and other members of its cytosolic complex, we treated HepG2 cells transfected with both V5-tagged CAR and CCRP for 24 hours with DMSO, TCPOBOP, MG132, or TCPOBOP combined with MG132. Consistent with our previous results, TCPOBOP alone decreased cytosolic CAR in the absence of CCRP, while in the presence of CCRP both CAR and CCRP levels were decreased by TCPOBOP (Fig 4A, *top panel*). However, in the presence of overexpressed CCRP, a portion of this decrease can be accounted for by the slight decrease in GAPDH protein (Fig. 4A, *bottom panel*). Immunoblotting analysis of protein levels for HSP 90, HSP 70, and HSP60 reveals that TCPOBOP caused very subtle changes in the levels of these chaperones. In contrast, MG132 increased levels of CAR, CCRP and all HSPs analyzed after factoring in a reduced level of GAPDH (Fig. 4A, *left panel* *s*). This is in contrast to effect of ALLN on multiple HSPs, where only HSP70 was induced due to an increase in both protein synthesis and half-life [40]. In the presence of MG132, TCPOBOP did not induce a reduction of CAR and CCRP, but this effect might have been masked due to MG132 increasing CCRP levels under these experimental conditions. Nevertheless, since MG132 caused HSP70 levels to increase together with CAR suggests that this chaperone might be a component of the CAR cytosolic complex.

To investigate this possibility, immunoprecipitation experiments using anti-HSP70 antibodies were performed on the same extracts analyzed in Figure 4A. In the absence of V5-tagged CCRP, CAR co-precipitated with HSP70 (Fig 4B, *top left panel*), providing first evidence that CAR interacts with HSP70 in the cytosol. Moreover, with TCPOBOP treatment, CAR was not co-precipitated with HSP70 indicating it had dissociated from HSP70 and possibly had undergone nuclear translocation. When CCRP was co-overexpressed with CAR, the amount of CCRP that co-precipitated with HSP70 and CAR was low (Fig 4B, *top right panel*) in spite of the high level of overexpressed CCRP in the cytosol (Fig 4A, *top panel*). CCRP and HSP70 are capable of interacting independent of CAR, due to CCRP possessing a J domain whose function is to bind HSP70 [41]. TCPOBOP treatment, while reducing CAR bound to HSP70, also caused a reduction in CCRP levels that was more apparent with longer exposure of the immunoblots shown in the top panels in Fig 4B (data not shown). In contrast, when cells were treated with MG132, both CCRP and CAR were co-precipitated with HSP70 and with a greater amount of CCRP co-precipitating compared to that in DMSO controls (Fig 4B, *top right panel*). These results demonstrate that proteasome inhibition stabilizes the CAR, CCRP and HSP70 complex. Interestingly, TCPOBOP co-treatment with MG132 did not produce a strong dissociation of CAR from HSP70 (Fig 4B, *top right panel*). In addition, HSP90 could also be detected in HSP70 immunoprecipitates. (Fig 4B, *bottom right panel*). Collectively, these data provide first evidence of HSP70 being an important component of the CAR cytosolic complex, and that HSP70 may regulate the levels of cytosolic CAR in conjunction with CCRP.

### Heat stress elevates cytoplasmic CAR in an HSP70-dependant manner

**Figure 5:**
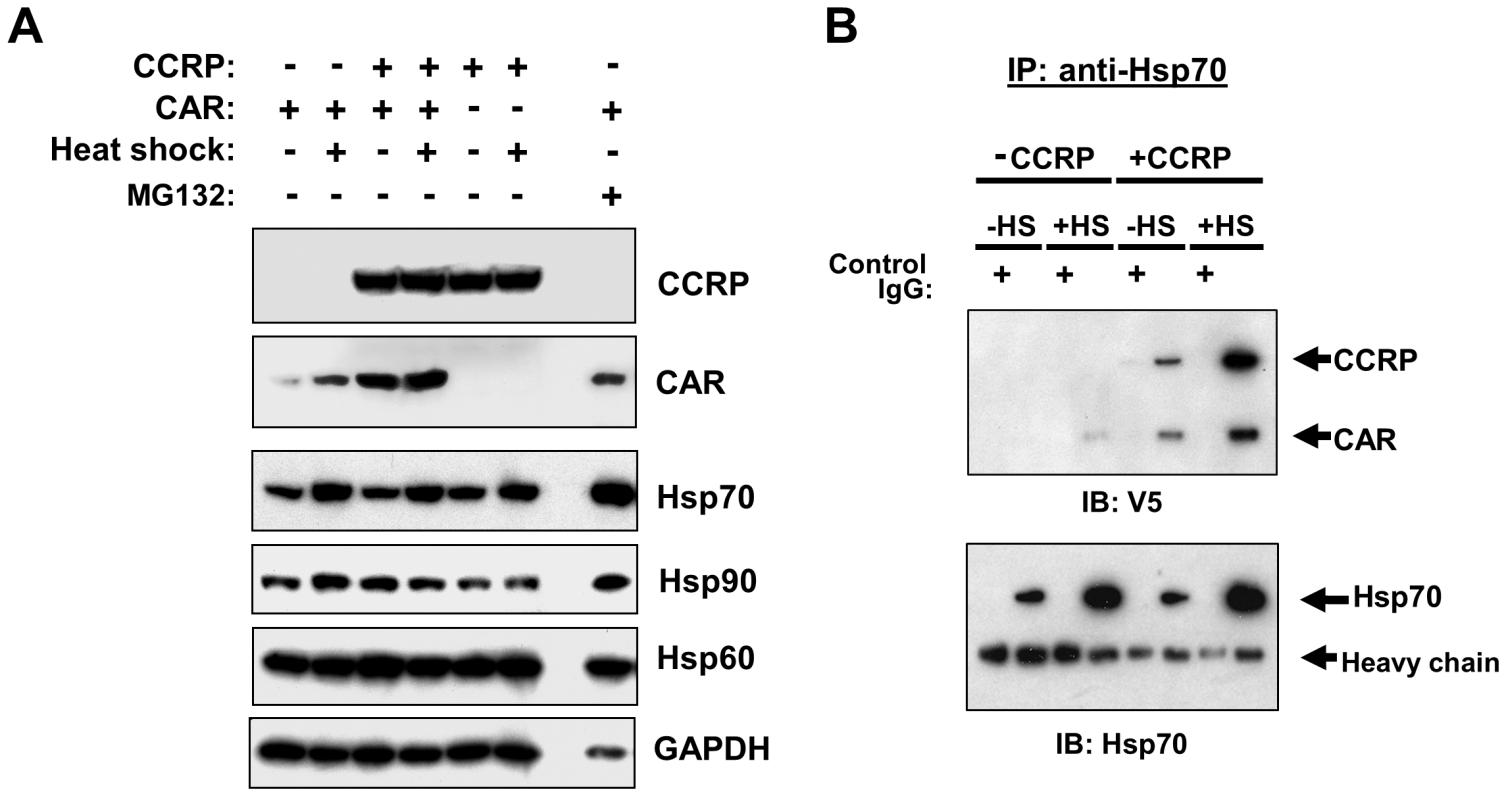
Thermal stress increases cytoplasmic CAR in an HSP70-dependent manner. Results shown are representative of three independent experiments. (A) HepG2 cells were cotransfected with V5-tagged CCRP (pcDNA3.1/V5-His-mCCRP, 0.3 µg) or empty vector and FLAG-tagged mCAR (pCR3-mCAR-FLAG, 3 µg) or empty vector. 18 hr after transfection, cells were incubated for 1 hr at 42°C or 37°C, followed by treatment at 37°C with DMSO (0.1% DMSO v/v) or MG132 (5 µM in 0.1%DMSO, final concentrations). Cells were then harvested and cytosolic extracts prepared and subjected to immunoblotting analysis with antibodies against the indicated proteins. (B) HepG2 cells were transfected with V5-tagged mouse CAR (pcDNA3.1/V5-His-mCAR, 3 µg) and mouse CCRP (pcDNA3.1/V5-His-mCCRP, 0.3 µg). 18 hr after transfection, cells were incubated for 1 hr at 42°C or 37°C, followed by treatment at 37°C with DMSO for 24 hr. Cell extracts were prepared and then subjected to immunoprecipitation analysis using anti-V5 or anti-IgG (as control) antibodies, followed by immunoblotting analysis using anti-V5 (top panel) and anti-HSP70 (bottom panel) antibodies.

To further establish a role for HSP70 in regulating levels of cytosolic CAR, we investigated whether modulation of HSP70 in ways other than treatment with MG132 will alter cytoplasmic CAR levels. As HSP70 is inducible by heat stress in HepG2 cells [42], [43], we overexpressed FLAG-tagged CAR with or without V5-tagged CCRP expression and then exposed cells to a 1-hour heat treatment at 42°C. In one study, this treatment resulted in maximum induction of HSP70 that was assessed 24 hours after heat shock [42]. In the 24 hours following heat stress, cells were treated with DMSO and cytosolic extracts prepared and subjected to immunoblotting analysis (Fig. 5 and Fig. S1). In the absence of CCRP co-overexpression, heat shock resulted in an increase in CAR levels, correlating with an increase in HSP70 levels; a modest increase in HSP90 protein and no changes in HSP60 levels were also found (Fig. 5A and Fig. S1). Heat shock caused similar effects on CAR and HSP70 levels in the presence of CCRP expression, and when factoring the slight decrease in GAPDH protein levels CCRP is also slightly elevated. In the absence of CAR overexpression, however, the level of CCRP was not changed. Interestingly, when comparing the effect of heat shock to the effect of MG132 treatment during the 24 hours after heat shock, both conditions elevated the level of CAR in the cytosol although MG132 has a greater CAR-elevating effect when factoring the decrease in GAPDH levels with MG132 treatment.

To determine whether HSP70 stabilizes CAR under conditions of thermal stress, immunoprecipitation analysis was performed. CAR co-precipitated with HSP70, and the amount of CAR bound to HSP70 increased in tandem with the elevation of HSP70 caused by heat shock. The same result was obtained with CCRP overexpression, with the added result that CCRP co-precipitated with HSP70 and CAR and that the amount of CCRP found in the complex correlates with HSP70 levels. These results indicate that changes in the level of HSP70, such as that induced by heat shock, regulate the level of both CAR and CCRP in the cytoplasm. Hence, since proteasomal inhibition causes changes in CAR and CCRP levels in the same direction as that caused by heat stress, and that both conditions cause elevations in HSP70 levels, we propose that HSP70 mediates the stabilization of both CAR and CCRP, and that HSP70 might play a role in CCRP ubiquitination.

### Attenuation of CAR ligand-induced transcriptional activation by heat stress

**Figure 6:**
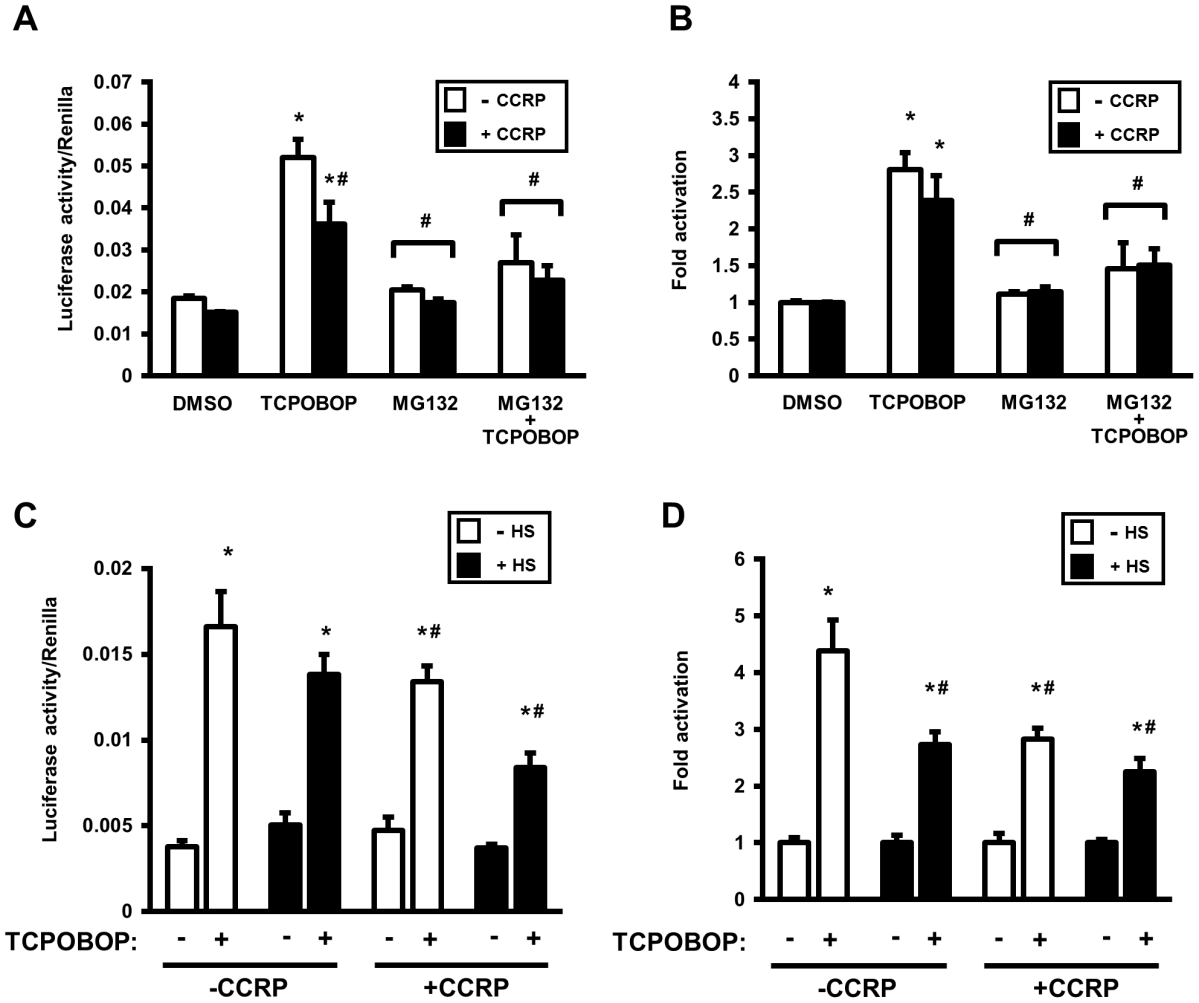
Thermal stress attenuates TCPOBOP-induced CAR transcriptional activation in HepG2 cells, similar to the effect of proteasomal inhibition. Luciferase activity of each sample was normalized to Renilla activity, and expressed as means ± SD of triplicate determinations. Shown is representative of three independent experiments. (A and B) Experiments were performed as described in Fig. 3, however empty vector or pcDNA3.1/V5-His-mCCRP was cotransfected with pcDNA3.1/V5-His-mCAR, -1.8-kb-luc, and phRL-tk (as normalization control). After transfection, cells were treated for 24 hr with DMSO (0.1% v/v), TCPOBOP (250 nM dissolved in 0.1% DMSO, final concentrations), MG132 (5 µM in 0.1% DMSO, final concentrations), or MG132 plus TCPOBOP, after which cells were harvested and luciferase activity measured. For fold-change determinations (B), each group was normalized to its corresponding DMSO-treated control (set as 1). Statistics are based on one-way ANOVA with post-hoc Tukey multiple comparisons test. *Significantly different (*p*<0.0001) compared to DMSO; and ^#^significantly different (*p*<0.002) compared to TCPOPOP without CCRP overexpression. (C and D) Experiments and data analysis were performed as in A and B, with an incubation step at 42°C for 1 hr for heat-shock-designated cells performed prior to 24 hr treatment with DMSO (0.1% v/v) or TCPOBOP (250 nM in 0.1% DMSO, final concentrations). For fold-change determinations (D), each group was normalized to its corresponding DMSO-treated control (set as 1). Statistics are based on one-way ANOVA with post-hoc Tukey multiple comparisons test. *Significantly different (*p*<0.0001) compared to corresponding DMSO control treatment; ^#^significantly different (*p*<0.001) to TCPOPOP alone (without both CCRP overexpression and heat shock).

As heat stress elevates cytosolic HSP70 levels, with concomitant elevation of CAR and to a smaller extent CCRP, we sought to determine whether ligand-activated CAR transcriptional activation is altered by heat stress. Firstly, it was important to establish the effect of increased cytosolic retention of CAR by means other than proteasomal inhibition. With the observations described earlier that CCRP overexpression reduces the ability of TCPOBOP to translocate accumulated CAR to the nucleus, TCPOBOP-induced CAR transcriptional activation was assessed by overexpressing mCCRP with mCAR in HepG2 cells and using the PBREM-containing -1.8-kb-luc reporter. As a control, experiments were performed using a -1.6-kb-luc reporter lacking the distal CYP2B6 phenobarbital responsive enhancer module (PBREM), and TCPOBOP-induced reporter activity was absent (data not shown). Normalized luciferase (Fig. 6A) and fold-change (Fig. 6B) activities are shown under the various conditions. TCPOBOP induced CAR transcriptional activity both in the absence and presence of overexpressed CCRP (Figs. 6A and 6B); CCRP overexpression attenuated slightly the induction by TCPOBOP. Treatment with MG132 robustly attenuated TCPOBOP-induced reporter activity (absolute and fold-change) irrespective of CCRP overexpression (Figs 6A and 6B), demonstrating again the potent effect of proteasome inhibition on CAR-mediated transcriptional activation.

The effect of a one-hour heat shock to induce HSP70 prior to TCPOBOP treatment was then assessed, using the −1.8 kb-luc reporter plasmid. Cells transfected with CAR with or without CCRP overexpression were treated with TCPOBOP for 24 hours following a 1-hour heat shock, and reporter activity was measured. Normalized luciferase (Fig. 6C) and fold-change (Fig 6D) activities are shown after treatment under the various conditions. TCPOBOP gave a robust induction of reporter activity, and adding heat shock attenuated this induction (Figs. 6C and 6D). CCRP overexpression attenuated TCPOBOP-induced reporter activation, and this was similar to the effect of heat shock (Figs. 6C and 6D). But interestingly, combining CCRP overexpression with heat shock gave further reduction in TCPOBOP-induced reporter activation (Figs. 6C and 6D). Overall, while both CCRP overexpression alone and heat shock alone caused a ∼36% attenuation of TCPOBOP-induced CAR transcriptional activation, the attenuation produced by the combination of CCRP overexpression and heat shock was similar to the effect of MG132 (∼50-60%) (Figs. 6B and 6D). These data provide further functional evidence for coordinated role of CCRP, HSP70 (through induction by heat shock), and the proteasome for regulating CAR in cells.

## Discussion

Insights into the mechanisms of CAR regulation have expanded considerably over the past decade. These mechanisms now include the activity of phosphatases (eg PP2A) and membrane-associated binding partners (eg R16A, Ect2), and more recently, scaffold proteins associated with RTKs such as RACK1. We now provide evidence for the role of ubiquitin-proteasome system in the regulation of CAR, and also have uncovered a role for HSP70 in this regulation. Our results strongly support that the CAR cytoplasmic partner CCRP, and not CAR itself, is ubiquitinated, thereby bringing CAR under regulation by the proteasome. We first observed that inhibiting the proteasome with MG132 leads to stabilization of CCRP levels. There was some experimental evidence for TCPOBOP-induced loss of CCRP levels in the absence of MG132 (Figure 4), however there was inconsistency between experiments attributable the very high levels of overexpressed CCRP in HepG2 cells which could not be titrated down. We also noted a significant amount of cytosolic CAR protein present when CCRP is overexpressed in HepG2 cells. Combining CCRP overexpression with MG132 treatment, we observed further increase in cytosolic levels of CAR, a result consistent with greater retention of synthesized CAR due to inhibition of CCRP degradation. Therefore, these findings suggest that in the presence of CAR activators, CCRP is ubiquitinated and consequently degraded by the proteasome, releasing CAR to translocate to the nucleus. Our finding of TCPOBOP-stimulated, CAR-dependent CCRP ubiquitination (Figure 2) is consistent with this activation mechanism.

The model of CCRP ubiquitination in CAR activation in HepG2 cells contrasts to that proposed in a recent report [44] using primary hepatocytes suggesting human CAR is ubiquitinated. MG132 concentrations used in experiments (0.1–10 uM) did not significantly differ from that used in our experiments (5 uM), and both primary hepatocytes and HepG2 cells possess intact ubiquitin-proteasome systems and express the endogenous HSPs. But the data in [44] did not necessarily support a conclusion that CAR is ubiquitinated. Firstly, there was a lack of detectable higher molecular weight-shifted species (each by ∼8–10 kD) of CAR. Second, the results of anti-FLAG immunoprecipitation experiments (anti-FLAG antibodies to pull down FLAG-tagged ubiquitin) may be explained as CAR co-precipitating with ubiquitinated protein. In our hands, purifying FLAG-tagged CAR then probing for Myc-tagged ubiquitin using anti-Myc antibodies gave no evidence for CAR being ubiquitinated (Figure 2). Most importantly,however, is that the above caveats and the results obtained in our hands do not exclude the possibility that CAR is itself ubiquitinated.

As CCRP is ubiquitinated in the absence to inducer treatment (Figure 2), CCRP must undergo a continuous turnover cycle. However, other processes may also establish CCRP levels and consequently influence CAR cytoplasmic levels and signaling. In terms of function, CCRP plays a role in fate decisions of unfolded proteins through interactions with HSP70 and HSP90 [45], [46]. It is unknown whether CCRP protein kinetics is subject to expression regulation as the HSPs mediated by heat shock factors (HSFs) [47]. However, ubiquitination has now been identified as one mechanism for regulating CCRP levels. It will be interesting to further investigate CCRP ubiquitination and its consequential effects on unfolded proteins and its interactions with HSP70 and HSP90. Insights could be obtained from experiments with HSP modulators (for example, geldanamycin), and by extension, assess the effects of HSP modulators on CCRP and consequences to CAR signaling.

**Figure 7:**
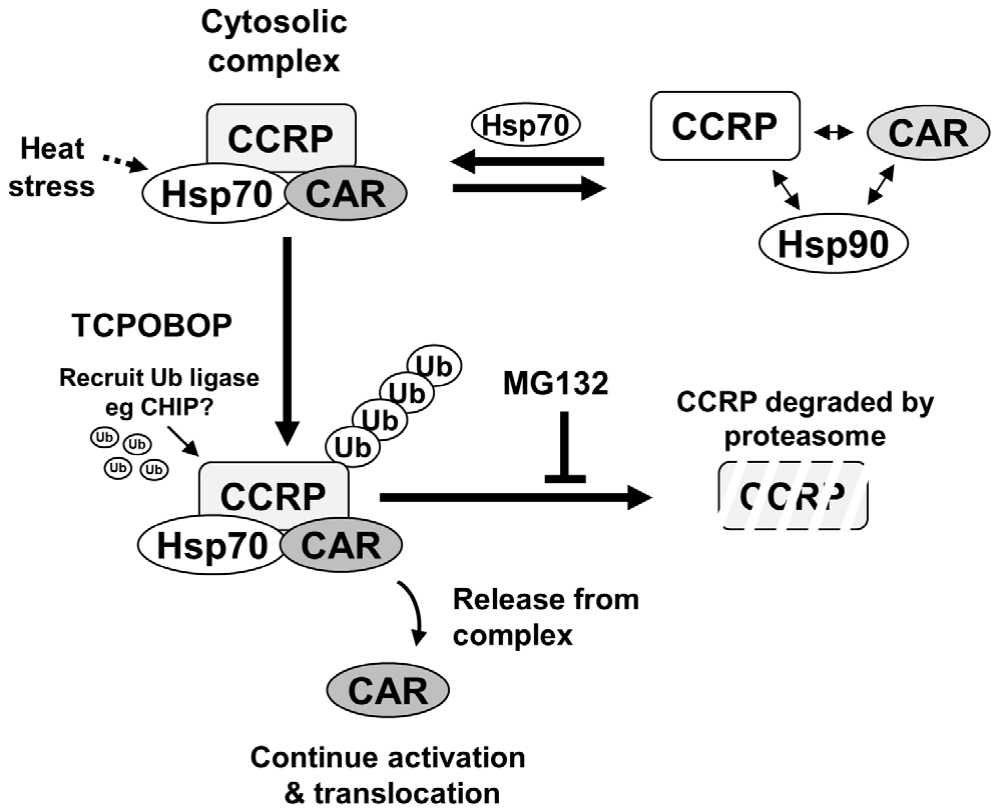
Proposed model for the regulation of CAR by the ubiquitin-proteasome system. An equilibrium between a stable CAR cytoplasmic complex (at top left) and a weaker associated complex exists (at top right), and HSP70 functions to favor the stable cytoplasmic complex. Activator treatment signals for ubiquitination of CCRP, resulting in its degradation by the proteasome. This results in the release of CAR from the cytosolic complex, followed by translocation into the nucleus.

These findings suggest a simple model for CAR retention and regulation by the ubiquitin-proteasomal system (Fig. 7). In our model, we propose that CAR exists in equilibrium between a complexed and dissociated state with its cytoplasmic partners. With increased CCRP expression and in concert with HSP70, a heteromeric protein complex that includes CAR, CCRP, and HSP70 is favored resulting in cytosolic stabilization of CAR. CCRP ubiquitination would therefore regulate the amount of stabilized CAR in the cytosol. Upon treatment with a CAR activator, CCRP ubiquitination and degradation would increase (over basal levels) resulting in the release of CAR and its subsequent translocation to the nucleus. Consistent with this model, increased cytoplasmic retention of CAR by proteasomal inhibition would reduce transcriptional activity, and this was indeed our observation and consistent with that observed for human CAR [44].

To strengthen the validity of our model, it was important to utilize a different means to increase cytoplasmic CAR and evaluate effects on CAR's transcriptional activity. In light of CAR interacting with HSPs, a simple approach would be to expose HepG2 cells expressing CAR and CCRP to thermal stress to induce HSP levels. We were able to demonstrate heat-shock induction of HSP70, and we observed that the HSP70 elevation correlated with an increase in cytosolic CAR. As it known that proteasomal inhibition also induces levels of certain cellular HSPs we tested the combination of both conditions. Indeed, we found that the increase in CAR levels by proteasomal inhibition was similar to the effect of heat stress, both correlating with increase in both cytosolic HSP70 and CAR and HSP70. The combination of both MG132 and heat shock treatments gave a much more robust elevation of cytosolic CAR. As we show that HSP70 strongly binds the CAR-CCRP complex, the increase in HSP70 by heat shock favors this stable complex and thus increasing cytoplasmic CAR levels. With an increase of CAR in the cytoplasm, it was predicted that both basal and maximum TCPOBOP-induced transcriptional activity would be elevated. However, we found that basal activity was not dramatically affected, and maximum TCPOBOP-induced transcriptional activity was reduced. This attenuation of TCPOBOP-induced transcriptional activity by heat shock in particular suggests that HSP70-mediated CAR retention is strong, to a degree that TCPOBOP at 250 nM concentration is less efficacious to activate CAR. That is, HSP70 also plays a crucial role to stabilize CAR in the cytoplasm, working in tandem with CCRP. Interestingly, HSP90 was detected in the CAR-HSP70-CCRP complex only in the presence of MG132; in the absence of MG132, HSP90 was not detectable in the complex, suggesting that HSP90 may regulate CAR levels and/or activity through a different mechanism. In summary, the results of this study support the model of CAR cytosolic sequestration by CCRP and HSP70, and upon activator treatment CCRP is ubiquitinated and degraded by the ubiquitin-proteasome system to release CAR to translocate to the nucleus. In the resting state, the same mechanism would serve to regulate the amount of CAR present in the cytoplasm.

Some considerations to our model remain to be addressed. First, the ubiquitin liagase(s) (E3s) responsible for CCRP ubiquitination need to be conclusively identified. We made attempts to address this through the use of *in vitro* CCRP ubiquitination assays with purified candidate E3s. Two enzymes, UFD2b, shown to form a protein-protein interaction with CCRP [48], and carboxyl-terminus of Hsc70-interacting protein (CHIP) [49], [50], were evaluated for their ability to ubiquitinate GST-tagged CCRP. However the inability to express UFD2b in *E. coli* and to obtain convincing results with purified CHIP prevented a determination of their ability to ubiquitinate CCRP. This does not preclude these E3s for ubiquitinating CCRP, in particular CHIP, owing to a large body of published evidence that it interacts with HSP70 and that it can regulate steroid nuclear receptor levels and function by directly ubiquitinating these receptors [51]–[54]. Furthermore, as we now show that HSP70 interacts with both CAR and CCRP, HSP70 may recruit CHIP to the CAR cytoplasmic complex to ubiquitinate CCRP to regulate cytoplasmic CAR levels. On the other hand, as UFD2b interacts directly with CCRP, it might function to ubiquitinate CCRP and hence regulate cytoplasmic CAR levels. Another possible mechanism is that CHIP and/or UFD2b may serve to ubiquitinate CCRP in the resting state, and upon CAR activator treatment additional E3(s) may function to increase CCRP ubiquitination for CAR release and translocation. For such E3-mediated mechanisms for CAR release upon activator treatment, it will be interesting how the binding of a CAR activator transduces into the signal to activate CCRP ubiquitination for CAR's release and nuclear translocation.

A second consideration is whether the ubiquitin-proteasomal system regulates CAR levels in the nucleus. This was attempted by using HepG2 cells stably expressing V5-tagged CAR (Ym17 cell line), as this cell line expresses CAR predominantly in the nucleus [33]. This cell line has been used extensively in studies examining CAR transcriptional activity and role of transcriptional coactivators/corepressors [55], [56]. When Ym17 cells were treated with MG132, CAR protein levels were elevated in the cytoplasm and not in the nucleus (data not shown). While this does not exclude a role of the ubiquitin-proteasomal system in regulating nuclear CAR levels, this result provides supplementary support for the ubiquitin-proteasomal system acting more prominently to regulate CAR levels in the cytoplasm and facilitate release from the cytosolic complex. However it is conceivable that, like for the steroid receptors ER and GR, the proteasome contributes to CAR-mediated transcription at the chromatin level, and is a possibility that merits further study.

In conclusion, we have identified two novel components of the molecular mechanisms for regulating CAR localization and function. These are: 1) CAR binding to HSP70; and 2) CCRP ubiquitination, inducible by CAR activators. We have also found that in addition to CCRP overexpression, heat shock and HSP70 induction elevates CAR in the cytoplasm, and similar to CCRP overexpression this sequesters CAR in the cytoplasm and attenuates CAR transcriptional activity. However, with the identification of TCPOBOP-induced CCRP ubiquitination, we have found a mechanism by which CAR translocation might be facilitated and/or enhanced. Identifying the E3 ligase catalyzing CCRP ubiquitination will be an important step. The combination of elevated HSP70 levels and with proper expression of the requisite E3(s) will undoubtedly enable the development of an *in vitro* cell culture system which recapitulates the *in vivo* characteristics of CAR and its activation by xenobiotics.

## Supporting Information

Figure S1 **HSP70 induction by heat shock elevates cytosolic CAR, similar to the effect of either CCRP overexpression or MG132 treatment.** HepG2 cells were cotransfected with V5-tagged CCRP (pcDNA3.1/V5-His-mCCRP, 0.3 µg) or empty vector and FLAG-tagged mCAR (pCR3-mCAR-FLAG, 3 µg) or empty vector. 18 hr after transfection, cells were incubated for 1 hr at 42°C or 37°C, followed by treatment at 37°C with DMSO (0.1% DMSO v/v; labeled “D”), TCPOBOP (250 nM dissolved in 0.1% DMSO, final concentration; labeled “TC”), or MG132 (5 µM in 0.1% DMSO, final concentration; labeled “MG). Cells were then harvested and cytosolic extracts prepared and subjected to immunoblotting analysis with antibodies against the indicated proteins. Results shown are representative of three independent experiments. With heat shock, CCRP levels were elevated together with HSP70 (*lanes 10-12 vs 4-6*), and CAR was concomitantly elevated (*lanes 10 vs 4*). The elevation of CAR with heat shock alone was equivalent to the effect of CCRP overexpression in the absence of heat shock (*lanes 7 vs 4*). The TCPOBOP-induced decrease of cytosolic CAR is maintained with CCRP overexpression and heat shock. Lastly, the combination of heat shock, CCRP overexpression and MG132 treatment resulted in the highest level of CAR in cytosolic extracts.
(TIF)
