# Supplementary figures and images for: Coordinated Regulation of Nuclear Receptor CAR by CCRP/DNAJC7, HSP70 and the Ubiquitin-Proteasome System
Source: PLoS One. 2014 May 2;9(5):e96092. doi: 10.1371/journal.pone.0096092 (PMC4008524; doi:10.1371/journal.pone.0096092)

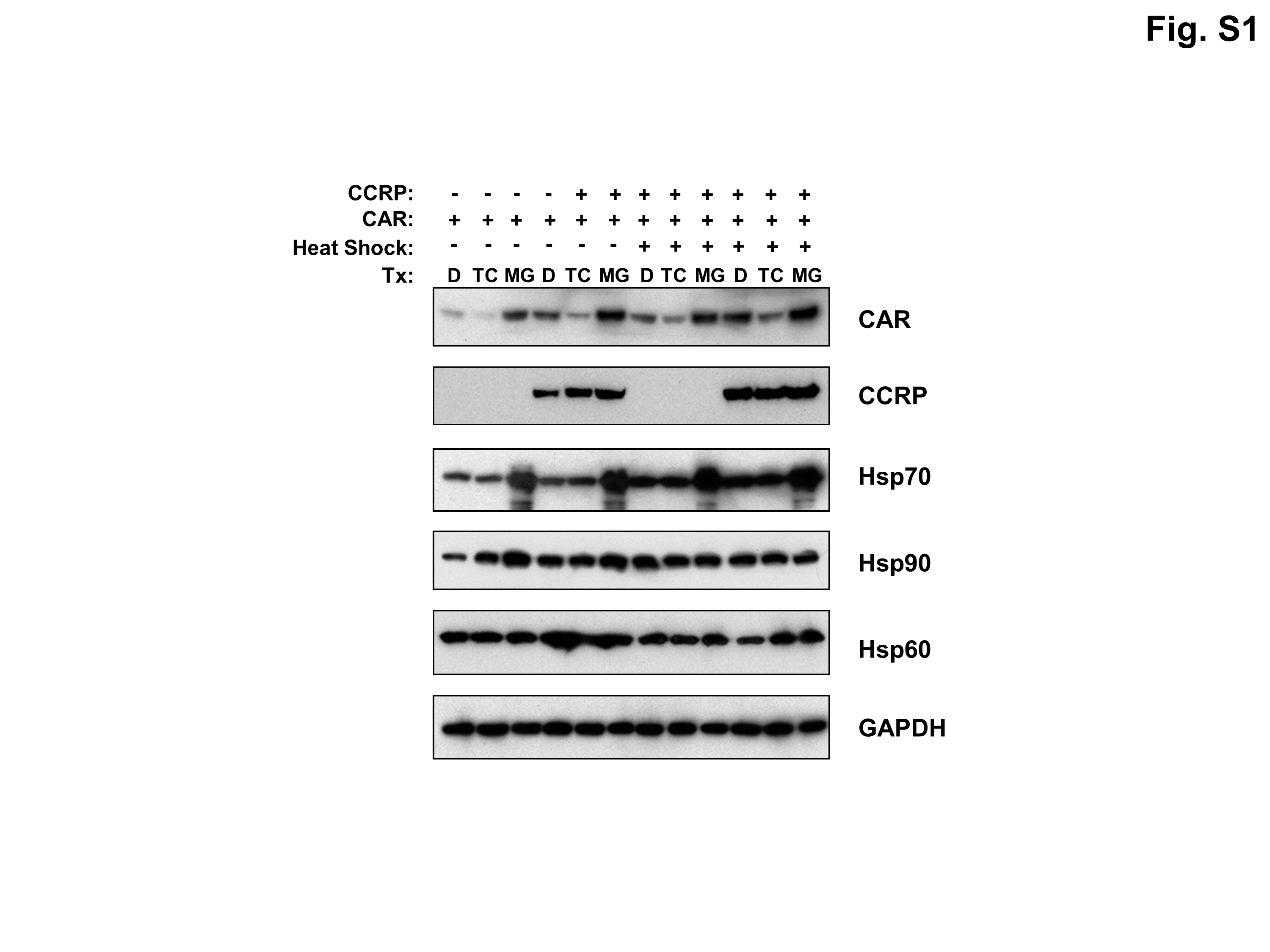

Supplement: Figure S1 — HSP70 induction by heat shock elevates cytosolic CAR, similar to the effect of either CCRP overexpression or MG132 treatment. HepG2 cells were cotransfected with V5-tagged CCRP (pcDNA3.1/V5-His-mCCRP, 0.3 µg) or empty vector and FLAG-tagged mCAR (pCR3-mCAR-FLAG, 3 µg) or empty vector. 18 hr after transfection, cells were incubated for 1 hr at 42°C or 37°C, followed by treatment at 37°C with DMSO (0.1% DMSO v/v; labeled “D”), TCPOBOP (250 nM dissolved in 0.1% DMSO, final concentration; labeled “TC”), or MG132 (5 µM in 0.1% DMSO, final concentration; labeled “MG). Cells were then harvested and cytosolic extracts prepared and subjected to immunoblotting analysis with antibodies against the indicated proteins. Results shown are representative of three independent experiments. With heat shock, CCRP levels were elevated together with HSP70 (lanes 10-12 vs 4-6), and CAR was concomitantly elevated (lanes 10 vs 4). The elevation of CAR with heat shock alone was equivalent to the effect of CCRP overexpression in the absence of heat shock (lanes 7 vs 4). The TCPOBOP-induced decrease of cytosolic CAR is maintained with CCRP overexpression and heat shock. Lastly, the combination of heat shock, CCRP overexpression and MG132 treatment resulted in the highest level of CAR in cytosolic extracts. (TIF) [file pone.0096092.s001.tif]
